# Adherence to Iron and Folic Acid Supplement and Its Associated Factors among Antenatal Care Attendant Mothers in Lay Armachiho Health Centers, Northwest, Ethiopia, 2017

**DOI:** 10.1155/2019/5863737

**Published:** 2019-06-02

**Authors:** Gashaw Agegnehu, Azeb Atenafu, Henok Dagne, Baye Dagnew

**Affiliations:** ^1^Tikl Dingay Health Center, Ethiopia; ^2^Department of Human Nutrition, Institute of Public Health, College of Medicine and Health Sciences, University of Gondar, Gondar 196, Ethiopia; ^3^Department of Environmental and Occupational Health and Safety, Institute of Public Health, College of Medicine and Health Sciences, University of Gondar, Gondar 196, Ethiopia; ^4^Department of Human Physiology, School of Medicine, University of Gondar, P.O. Box 196, Gondar, Ethiopia

## Abstract

**Background:**

Iron deficiency is the leading nutrient deficiency globally affecting the lives of more than two billion people worldwide. Pregnant women are at higher risk of iron and folic acid deficiency due to lack of iron and folic acid or due to poor adherence. Adherence to iron and folic acid supplement is taking 65% or more of the recommended supplement, equivalent to taking the supplement at least 4 days a week during 3 months period using recording, reporting, and checking cards.

**Objective:**

The current study aimed at assessing adherence to iron and folic acid supplement during pregnancy and its associated factors among pregnant women attending antenatal care.

**Methods:**

Institution based cross-sectional study was conducted from February to March 2017. Systematic random sampling technique was used to select the study subjects. Data were collected using structured and pretested interviewer-administered questionnaire. Bivariable and multivariable logistic regression analysis were used to identify factors associated with adherence to iron and folic acid supplement among pregnant women. Adjusted odds ratio (AOR) with a 95% confidence interval (CI) was used to display the level of significance. Variables with a p-value less than 0.05 had been considered statistically significant.

**Result:**

Adherence to iron and folic acid in the current study was 28.7% with 95% CI (24.3, 33.6%). Educational status of mothers (AOR= 9.27 (95%CI: 2.47, 34.71)), educational status of husband (AOR= 0.31(95% CI: 0.11,0.88)), family size of four (AOR=3.70(95%CI: 1.08,12.76)), family size of five and above (AOR= 4.88(95% CI: 1.20, 19.85)), mothers who had 2500-3500 Ethiopian birr household average monthly income (AOR= 0.46(95% CI: 0.24,0.89)), mothers who had registered at 17-24 weeks of gestation (AOR=0.40(95% CI: 0.22-0.74)) and registered at 25-28 weeks (AOR=0.20(95% CI 0.10, 0.41)), and mothers who had collected their iron and folic acid starting at first visit at first month of pregnancy (AOR= 2.42(95% CI:1.05, 5.58)) had significant association with iron and folic acid adherence.

**Conclusion and Recommendation:**

Adherence of iron and folic acid was only 28.7% in the current study. Maternal and husband education status, family size, registration time, economic status, and first visit in the first month with duration of iron and folic acid taken were factors significantly associated with adherence to iron and folic acid supplement. Therefore, anaemia prevention strategy via improved iron and folic acid supplement adherence should comprise strategies of educating pregnant mothers, improving economic status, and early antenatal care (ANC) registration that can improve adherence to iron and folic acid supplement.

## 1. Introduction

Iron deficiency is the leading single most important nutrient deficiency globally which affects the lives of more than two billion people, accounting for over 30% of global population, the highest being in developing countries [1]. Pregnant women, postpartum women, and children aged 6-24 months are usually the most affected groups [2, 3]. Pregnant women are at high risk of iron and folic acid deficiency due to increased nutrient requirement during pregnancy [4, 5]. In Ethiopia, 17% women with reproductive age and 22% of pregnant women are estimated to be anaemic [6, 7]. According to the WHO and Ethiopia's national guidelines for control and prevention of micronutrient deficiencies, all pregnant women should receive and consume a standard dose of 60 mg iron and 400 *μ*g folic acid daily for 6 months starting from first month of pregnancy or at the time of their first antenatal visit and three months of postnatal period. Iron and folic acid supplementation are required to balance increased physiological demand during puberty, pregnancy, and lactation [2, 3, 8, 9].

Iron is a trace mineral that is important for fetal growth and development. Iron is found in red blood cells to carry oxygen needed throughout the body. It is also essential for normal neuronal development. Folic acid is another important micronutrient used in the synthesis of neurotransmitters and particularly during early pregnancy. It has an essential role in synthesizing DNA during organogenesis [10].

The high physiological requirement for iron and folic acid in pregnancy is difficult to meet with the usual diet. Therefore, pregnant women should routinely receive iron and folic acid supplements. Adherence to a medication regimen is generally defined as the extent to which patients take medications as prescribed by their health care providers [11]. Adherence rates for individual patients are usually reported as the percentage of the prescribed doses of the medication actually taken by the patient over a specified period [12].

One in every three women had anaemia while one in every two had iron and folic acid deficiency, indicating that both folic acid and iron deficiencies constitute the major micronutrient deficiencies in Ethiopian women [4]. Iron and folic acid deficiency is a serious public health issue due to its high potential negative consequences. It can lead to several adverse outcomes including low birth weight, preterm delivery, stillbirth, and maternal and neonatal mortality. Infants are among the vulnerable groups of iron/folic acid deficiency. Since there is a link between maternal and neonatal iron status, interventions on infant alone are insufficient to decrease infant iron and folic acid status. Oral iron and folic acid supplementation is a feasible and cost-effective strategy in the prevention and control of iron and folic acid deficiency anaemia [13].

The risk of death decreases by 24% and 1.8 million deaths in children aged 28 days to 10 years will be avoided for each increase in 1 g/dl haemoglobin. Simple IFAS strategies are feasible ways to attain such increments in haemoglobin [13]. Early neonatal death was reduced by 57% in Nepal and 45% in Pakistan when more than 90 tablets are taken and started at or before fifth months of pregnancy. Similarly, the risk was decreased by more than half in those study participants who took IFAS during pregnancy [14]. Currently, iron and folic acid supplementation is the main strategy for anaemia control and prevention in Ethiopia [7, 9]. While many developing countries including Ethiopia are now implementing iron folic acid supplementation (IFAS) through antenatal care programs, only a few countries have reported significant improvement in IFAS adherence and control and prevention of anaemia [7, 15, 16].

**Figure 1:**
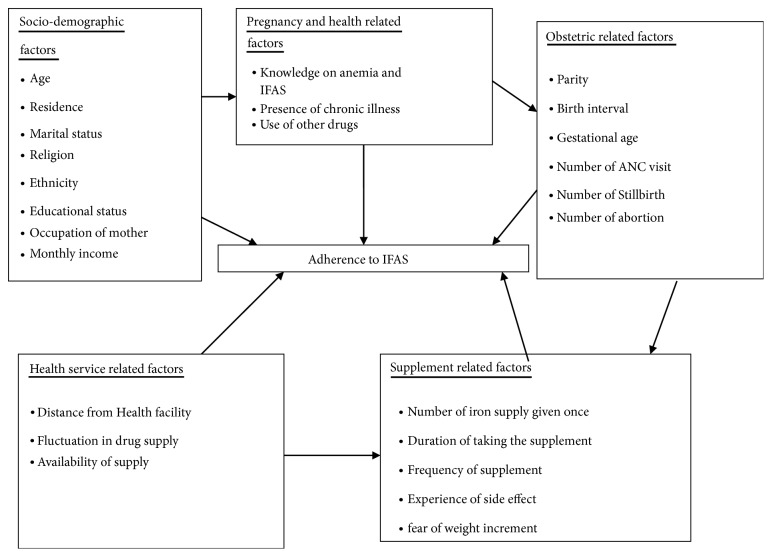
Conceptual framework adapted from different literature review [21, 24, 26].

This low achievement may be related to poor access and lower level of antenatal care service utilization, inadequate IFAS, poor counselling, poor knowledge about anaemia, and certain bad believes regarding IFAS [7]. However, many studies suggested that low level of maternal adherence to the regimen is the main reason for the ineffectiveness of the anemia prevention and control strategy [11, 15]. There is little feedback about the effectiveness of iron and folic acid supplement countrywide. Women may have low adherence to IFAS due to perceived side effects [17–19], forgetfulness [17, 18, 20, 21], and lack of access to IFAS supplement [11]. The factors associated with adherence to IFAS reported by various studies [18, 20–22] are not similar and there is a lack of information regarding the prevalence and associated factors of IFAS in the study area. In addition, previous studies mostly focused on adherence to IFAS and associated factors among pregnant women at community level where it lacks assessment of organizational related factors. Different sociodemographic, pregnancy and obstetric related, health service, and supplement related factors may contribute to adherence to IFAS (Figure 1). The level of education, the cultural practices, the strength of health service, and familial support vary from place to place. The current study focuses on assessment adherence and associated factors of IFAS among pregnant women in Lay Armachiho district health centers, Northwest, Ethiopia.

## 2. Methods

### 2.1. Study Design and Settings

An institution based cross-sectional study design was employed from February to March 2017 in Lay Armachiho district Health Centers. Lay Armachiho district is one of 22 districts in North Gondar administrative zone and is located at a distance of 769 *km* from Addis Ababa, 202 *km* from Bahirdar, and 22 *km* from Gondar administrative city. It encompasses about 129,272 *km*^2^ area of land and is characterized by diverse climate and topography with a marked difference in the climate conditions. Most of the area is on the highland plateau and is characterized by rugged mountains, hills, and plateaus. Hence, the district has varied landscapes composed of steep fault escarpments and nearly mountainous and erodible landforms but draught did not occurr and no shortage of rain in a rainy season. It is located in Northwest of Gondar, Ethiopia and bordered by Wogera, Sanja, Chillga, Dembia, and North Gondar administrative city. According to figures from the Central Statistical Agency, 2007, the district had an estimated population of 140417, of whom 70911 are females. Estimated pregnant women are 5,616. There are 6 health centers, 26 health posts, 6 junior private clinics, 1 medium private clinic, and 2 private drug venders providing health services in the district [23].

### 2.2. Sample Size Determination

The sample size was calculated using single population proportion formula with the following assumptions: p (Proportion of Adherence to IFAS = 55.5% [24], 95% confidence interval, and 5% margin of error (d)).

(1) nzα/22p1−pd2=1.9620.5551−0.5550.052=380

Considering, a 10% non-response rate, the final sample size was 418.

### 2.3. Sampling Procedures and Sampling Technique

There were a total of 838 pregnant women who fulfilled eligibility criteria (all pregnant mothers who were taking IFAS during their 2nd, 3rd, and 4th ANC follow-up were included in the study but mothers who were seriously ill at the time of data collection and pregnant mothers at the first trimester were excluded) during the study period in Lay Armachiho district, 2017. Using baseline information, a proportional number of study subjects were selected from each health center. The interval was determined as k = N/n, where k is the interval of sampling, N is total number of population, and n is the number of sample. With the total number of pregnant women N = 838 and the minimum sample size being n = 418, an interval of k = N/n = 838/418= 2. The first study participants were selected using lottery method and then every 2nd unit from ANC registration book was taken as study subject. Therefore, systematic random sampling technique was used to include a sample of 418 study participants.

### 2.4. Data Collection Procedures

A structured questionnaire was used to collect data. The questionnaire was pretested over 30 women out of the study area in an institution with similar characteristics prior to the actual data collection. Six BSc Nurses (worked as data collectors) and two BSc Public Health professionals (worked as supervisors) were involved in the data collection process. Training was given for the data collectors and supervisors. The overall interview process was supervised by supervisors. The collected data were checked by the data collectors immediately after finalizing the questionnaire before they left the health centers. Supervisors daily checked the completeness and consistency of information collected.

### 2.5. Operational Definition

Adherence to IFAS is the outcome variable in this study. Pregnant mothers are said to be “adherent ” to IFAS if they took 65% or more of the recommended supplement, equivalent to taking the supplement at least 4 days a week during 3 months of period using recording, reporting, and checking their cards [25]. Early registration to ANC clinic was measured as those pregnant women who visit the ANC clinic before 16 weeks of gestation [26]. Knowledge of anaemia was measured by asking 6 knowledge questions whether respondents know the illness called anemia, causes of anemia, and consequences of anemia during pregnancy and identify who is more susceptible to anemia and whether they know methods of anemia prevention during pregnancy. Mothers who score median and more questions on anaemia are said to be knowledgeable [21]. Knowledge about IFAS was measured by asking 8 knowledge questions about IFAS (regarding knowledge of Iron/folate drug, health benefit of IFAS for the fetus and child, whether they believe taking IFAS has risks, and whether they know for how long they should take the IFAS). Those who score median and above on questions prepared to assess comprehensive knowledge of IFAS of the respondents were considered as having “good knowledge” and those who scored below the median were having “poor knowledge” [26]. Total knowledge for both variables together was computed and respondents who score mean and above mean of the knowledge questions were considered as knowledgeable.

### 2.6. Data Management and Statistical Analysis

Data were entered using EPI-INFO version 3.5.1 and exported into SPSS version 20 for analysis. For most variables, data were presented by frequencies and percentages. Bivariable logistic regression analysis was used to explore candidate variables for the multivariable logistic regression analysis and variables with p-value less than 0.2 during the bivariable analysis were then analyzed by multivariable logistic regression for controlling the possible effect of confounders and finally variables which had significant association with IFAS adherence were identified on the basis of AOR with 95% CI and p < 0.05. Hosmer and Lemeshow goodness of fit test was used to check model fitness. Variance inflation factor (VIF) was also used to test interactions between variables.

## 3. Results

### 3.1. Sociodemographic Information

A total of 418 study participants were included in this study with 100% response rate. The mean age of respondents was 27.2 (SD±6.3) years. Majority of respondents 312 (74.6%) were rural dwellers. Four hundred fifteen (99.3%) of the study subjects were followers of Orthodox Christianity. The majority (83%) of the study subjects were housewives (Table 1).

### 3.2. Pregnancy, Obstetric, and Health-Related Factors of Respondents

Majority of respondents (72.2%) had less than three times ANC visit. Among the respondents, 124 (29.7%) had started ANC during the first trimester (before 16 weeks of gestation) (Table 2).

### 3.3. Respondents Knowledge of Anaemia and Benefit of IFAS

Less than one-fifth of respondents 57 (13.6%) were knowledgeable about anaemia and IFAS, while 361 (86.4%) of respondents were not knowledgeable about anaemia and IFAS (Table 3).

### 3.4. Health Service-Related Factors of Respondents

Only 4 study participants have used iron and folic acid for greater than three months (90 tablets). Among the respondents, 80% were provided health education about IFAS (Table 4).

### 3.5. The Adherence to IFAS

From a total of 418 participants included in this study, only 120 (28.7%) 95% CI (24.3, 33.6%) participants adhered to IFAS. The reason that most mothers reported for not adhering to IFAS were forgetfulness 167 (40%), pill burden 21 (5%), side effects of the drugs 207 (49.5%), epigastric burning sensation (gastritis) 206 (49.3%), unpleasant taste of IFAS 101 (24.2%), and vomiting 166 (39.7%).

### 3.6. Factors Associated with Adherence to IFAS

Bivariable logistic regression was used to choose variables for the final model on the basis of p-value less than 0.2. Place of residence, educational status of mothers, educational status of husband, number of pregnancy, number of delivery, knowledge on anaemia and IFAS, occupation of mother, occupation of husband, family size, early registration, average household monthly income, disease confirmed by physician like gastritis, treatment of disease, and first visit at first month were variables selected for the final model. Variance inflation factor (VIF) was calculated considering one independent variable as the dependent variable turn by turn to test interactions between variables to minimize effect of multicollinearity. The test result showed that VIF for all variables was below 3, the threshold for collinearity diagnostics. This showed that there is no multicollinearity effect between independent variables.

Table 5 shows variables associated with adherence to IFAS. Adherence to IFAS was significantly associated with the educational status of participants. The probability of adherence to IFAS was 9.27 times higher among participants whose educational status was primary education and above [AOR= 9.27, 95%CI= (2.47, 34.71)] as compared to study participants who cannot read and write.

About 69% of study participants whose husbands have an educational status above primary education were less likely adhered to IFAS as compared to those study subjects whose husbands cannot read and write [AOR= 0.31, 95% CI = (0.11, 0.89)]. The probability of IFAS adherence was 3.7 times higher among participants who have a family size of four as compared to those having only two family members [AOR=3.70, 95% CI = (1.08,12.76)]. Adherence of IFAS among study participants with five and above family members was 4.88 times higher than those only with 2 family members [AOR=4.88, 95% CI = (1.20, 19.85].

As revealed by this study, adherence to IFAS was associated with average monthly household income. The study participants whose average monthly income is 2501 to 3500 ETB were less likely adhered to IFAS compared to those who earned 500 to 2500 ETB [AOR= 0.46, 95% CI = (0.24,0.90)]. Participants who had registered 17-24 weeks of gestation [AOR=0.40, 95% CI (0.22-0.74)] and participants who had registered at 25-28 weeks [AOR=0.20, 95% CI (0.10, 0.41)] were less likely to adhere to IFAS. The probability of adherence to IFAS was 2.42 times higher among study participants collecting their pill at first visit at first month of pregnancy with duration of pill used [AOR= 2.42, 95% CI (1.05, 5.58)].

## 4. Discussion

Pregnant women are prone to iron deficiency anemia. Iron and folic acid supplementation has been a major strategy to reduce iron/folic acid deficiency anemia. However, issues of poor adherence remain a challenge. Adherence to IFAS found in this study was 28.7% which is lower than studies conducted in Cambodia [14], Indonesia [13], studies done among pregnant women in Eritrean refugee camps, Northern Ethiopia [19], among pregnant women attending ANC at University of Gondar hospital [18], Governmental Health Centers in Akaki Kality Sub City [22], among pastoralist pregnant women in Burji districts, Southern Ethiopia [17], among pregnant woman in west Dembia [27], and among pregnant women attending antenatal care follow up at Debre Tabor General hospital, Ethiopia [28]. It was similar to the adherence reported for rural women by Gebre et al. [26]. But the adherence in the current study is higher than results from studies conducted in Kenya [29], Goba, Ethiopia [24], a study done in 8 rural districts of Ethiopia [21], and a study conducted in Mecha district in Amhara region [30].

This difference could be due to the differences in the socioeconomic status of the study populations and the time of the study, the data collection tool used, and the difference in the study setting. This also might be due to differences in training level of health care professionals regarding IFAS and the professional skill of teaching mothers regarding the health benefit of IFAS and standard of the health care institution in the different level of health care facilities as this study was conducted on health centers. This rationale is supported by several studies as in well-organized set-ups adequate counselling and sustainable product availability is better [27].

This study depicted that the educational status of the mother was significantly associated with adherence to IFAS. The adherence was higher among mothers who have completed primary education and above.

This finding is in line with the findings of other studies in Ethiopia [17, 22, 30] and India [31]. Education is more likely to enhance female awareness of micronutrient deficiency and ways to overcome these deficiencies. Overall, educated women have a greater ability to stick to health care inputs such as IFA which offer better care for both the infant and the mother. A husband who completed primary education and above was less likely to promote adherence of IFAS. This result is inconsistent with a study conducted in Indonesia where both mothers and husbands had promoted and enhanced adherence status of pregnant women equally [32]. This might be due to low autonomy of mothers on their own health care in Indonesia [32]. In the current study mothers are autonomous on utilization of IFAS without the consent of their husbands.

Household average monthly income was associated with IFAS. The current study has revealed that those participants with lower family income are less likely to adhere to IFAS compared to those with higher family income. This was consistent with a study conducted in Kenya [29]. Family size was significantly associated with adherence of IFAS. This might be because those participants who had multiparity would have medical advice, health education, counselling, and access to more information about the benefits of IFAS during pregnancy and early pregnancy than nulliparous participants.

Early time of registration was significantly associated with IFAS adherence. This finding is consistent with a study done in Kenya [29].

Finally, this study is not without limitation. The gold standard method of measuring adherence like electronic and pills counting method was not used. The cross-sectional nature of the study does not allow establishing a strong cause-effect relationship. The use of larger sample size and pretested questionnaire made this study generalizable and the reliability was checked and found to be good.

## 5. Conclusion/Recommendations 

The adherence of IFAS among antenatal care attendant women in Lay Armachiho district health centers was low and significant proportion of women do not adhere to IFAS. Educational status of participants, educational status of husband, family size, household average monthly income, registration time, and tablet collection time were identified as factors associated with adherence to IFAS. Educating pregnant mothers, improving economic status, and early ANC registration can improve adherence to IFAS. Professionals at the health centers should strive a lot to increase the adherence of pregnant women on IFAS. There are many barriers that hinder the pregnant women from adherence to IFAS including forgetfulness and fear of side effects of the drugs. To minimize or prevent forgetfulness to take the drug, health professionals should counsel the women with strategies such as correlating taking the drugs with natural events. Regarding fear of side effects, health professionals should deliver counseling and make the pregnant women aware that side effects are minor as compared with the advantage of taking IFAS, and if major side effects occurred, they should go to health facilities for better management.

## Figures and Tables

**Table 1:** Sociodemographic factors of respondents, Lay Armachiho district Health Centers, Northwest Ethiopia, 2017 (n=418).

Variable	Categories	Frequency (n)	Percent (%)
Age	17- 25	211	50.5
	26- 35	155	37.1
	36- 45	52	12.4

Residence	Rural	312	74.6
	Urban	106	24.4

Religion	Orthodox	415	99.3
	Muslim	3	0.7

Ethnicity	Kemant	380	90.9
	Amhara	38	9.1

Educational status of mother	Cannot read and write	148	35.4
	Can read and write	188	45
	Completed primary and above	82	19.6

Educational status of husband	Cannot read and write	152	36.4
	Can read and write	118	24.6
	Completed primary and above	148	35.4

Occupation of mother	Housewife	347	83
	Employed	71	17

Occupation of husband	Employed	66	15.8
	Farmers	352	84.4

Family size	Two	101	24.2
	Three	77	18.4
	Four	64	15.3
	Five and above	176	42.1

Monthly income (ETB^*∗*^)	500- 2500	165	39.5
	2501- 3500	161	38.5
	>3500	92	22

*∗∗*Knowledge about IFAS and anaemia	Knowledgeable	57	13.6
	Non-knowledgeable	361	86.4

*∗* ETB: Ethiopian birr; ETB = 24 USD; *∗∗* knowledgeable means those who have correctly answered above the mean of knowledge questions regarding anemia and IFAS.

**Table 2:** Pregnancy, obstetric, and health-related factors of respondents, Lay Armachiho district Health Centers, Northwest Ethiopia, 2017 (n=418).

Variable	Categories	Frequency (n)	Percent (%)
Gravidity	One	114	27.3
	Two- Five	219	52.4
	Six- Ten	85	20.3

Parity	Zero	117	28
	One- five	257	61.5
	Six- Eight	44	10.5

History of stillbirth	Yes	22	5.3
	No	396	94.3

Number of stillbirth	One	18	4.3
	Two or more	4	0 .96

History of abortion	Yes	39	9.3
	No	379	90.7

Number of abortion	One	29	6.9
	Two or more	10	2.4

ANC follow up	Yes	415	99.3
	No	3	0.7

Time of ANC registration	0- 16 weeks	124	29.7
	17- 24weeks	160	38.3
	25- 28 weeks	134	32.1

**Table 3:** Respondents' knowledge of anaemia and benefit of IFAS in Lay Armachiho district Health Centers, Northwest Ethiopia, 2017 (n=418).

Variables		Frequency (n)	Percent (%)
Knowledge on anaemia and IFAS	Good	57	13.6
	Poor	361	86.4

The source of information	Health professionals	310	74.2
	Mass media	41	9.8
	Friends	21	5

Iron/and folic acid currently taking	Yes	403	96.4
	No	15	3.6

Duration of iron and folic acid used	1 month (30 Tablets)	162	38.8
	2 month (60 Tablets)	140	33.5
	3 month (90 Tablets)	116	27.8

**Table 4:** Health service-related factors of respondents, Lay Armachiho district health centers, Northwest, Ethiopia, 2017 (n=418).

Variable	Categories	Frequency (n)	Percent (%)
Health education delivered during collection of drugs about	Use of drugs	335	80
	Methods used	221	52.9
	Side effects	181	43.3
	Appointment	261	62.4

Tablets collected in each follow up	30 tablets	396	94.7
	60 tablets	7	1.7

*∗*Problem faced in health centre during health care delivery	Yes	24	5.7
	No	377	90.4

*∗* Respondents were asked if they encountered problems such as shortage of IFAS, long waiting time, poor communication that hindered them from taking the IFAS.

**Table 5:** Factor associated with adherence Lay Armachiho district Health Centers, Northwest Ethiopia, 2017 (n=418).

Variables	Category	Adherence status of respondents		COR(95% CI)	AOR(95% CI)
		Adhered	Non-adhered		
Residence	Rural	80(19.1)	232(55.5)	1	1
	Urban	40(9.6)	66(15.8)	0.57(0.36, 0.91)	0.60(0.27, 1.30)

Educational status of mother	cannot read and write	27(6.5)	121(28.95)	1	1
	Read and write	43(10.3)	145(34.7)	0.57(0.44, 1.29)	1.34(0.64, 3.05)
	Completed primary and above	50(12)	32(7.7)	5.27(3.01,9.22)	9.27(2.47,34.71) *∗*

Educational status of husband	Cannot read and write	36(8.6)	116(27.8)	1	1
	Read and write	28(6.7)	90(21.5)	0.10(0.57, 1.76)	0.78(0.38, 1.61)
	Completed primary and above	56(13.4)	92(22)	1.96(1.14, 3.35)	0.31(0.11-0.88) *∗*

Family size	Two	19(4.5)	82(19.6)	1	1
	Three	49(11.7)	127(30.4)	2.33(1.18,4.62)	2.33(0.86, 6.32)
	Four	27(6.5)	50(12)	2.77(1.36,5.62)	3.70(1.08, 12.76) *∗*
	Five and above	25(6)	39(9.3)	1.67(0.92, 3.03)	4.88(0.90, 4.80)

Occupation of mother	House wife	81(19.4)	266(63.6)	1	1
	Employed	39(9.3)	32(7.7)	4.00(2.36,6.80)	1.13(0.46,2.79)

Occupation of husband	Employed	39(9.3)	27(6.5)	1	1
	Farmer	81(19.4)	271(64.8)	0.63(0.24, 1.67)	0.66(0.25, 1.72)

Monthly income	500-2500 birr	28(6.7)	133(31.8)	1	1
	2501-3500 birr	40(9.6)	125(29.9)	0.66(0.38,1.13)	0.46(0.24,0.89) *∗*
	>3500 birr	52(12.4)	40(9.6)	4.06(2.34,7.00)	2.08(0.90,4.79)

Gravidity	One	28(6.7)	86(20.6)	1	1
	Two	46(11)	126(30.1)	1.76(0.95,3.26)	0.86(0.06,12.49)
	Three	15(3.6)	32(7.7)	1.44(0.86,3.04)	1.29(0.06,28.35)
	Four and above	31(7.4)	54(12.9)	1.12(0.65,1.93)	2.58(0.11,61.09)

Parity	Zero	28(6.7)	88(21.1)	1	1
	One	45(10.8)	124(29.7)	1.94(1.06, 3.55)	0.86(0.07, 11.32)
	Two	13(3.1)	55(13.2)	1.32(0.07,2.86)	0.61(0.03,12.78)
	Four and above	34(7.1)	31(7.4)	1.14(0.66,1.97)	0.22(0.01, 4.46)

Early registration	0-16weeks	55(13.2)	69(16.5)	1	1
	17- 24 weeks	44(10.5)	116(27.8)	0.48(0.29, 0.78)	0.40(0.22, 0.74) *∗*
	25- 28 weeks	21(5)	113(27)	0.66(0.13, 0.51)	0.20( 0.10,0.41) *∗∗*

Duration of pill taken	3months	100(23.9)	283(67.7)	1	1
	>3months	20(4.8)	15(3.4)	3.77(1.86, 7.66)	2.42(1.05, 5.58) *∗*

Health problem	Yes	3	26	1	1
	No	117	272	3.73(1.12,12.56)	1.32(0.12,14.64)

Treatment for health problem	Yes	2	22	1	1
	No	118	276	4.70(1.09, 20.32)	1.86(0.11, 33.18)

Knowledge on anemia and IFAS	Good	12(2.9)	45(10.8)	1	1
	Poor	108(25.8)	253(60,5)	0.63(0.32, 1.23)	0.77(0.36, 1.64)

*Note.∗∗* significant at P-value < 0.001; *∗* significant at P-value <0.05; Hosmer and Lemeshow test = 0.434 showed that the model fit well.

## Data Availability

Data will be made available upon request the primary author.

## List of Abbreviations

ANC: Antenatal care
AOR: Adjusted Odds Ratio
CI: Confidence interval
COR: Crude Odds Ratio
IFAS: Iron folic acid supplementation
Km: Kilometer
Km^2^: Square kilometers
mg: Milligram
SPSS: Statistical Package for Social Sciences
VIF: Variance inflation factor
WHO: World Health Organization
*μ*g: Microgram.
